# Genome Sequence of the Extremely Acidophilic Fungus Acidomyces richmondensis FRIK2901

**DOI:** 10.1128/MRA.01314-18

**Published:** 2018-10-25

**Authors:** Matthew D. Rosienski, Mi-Kyung Lee, Jae-Hyuk Yu, Charles W. Kaspar, John G. Gibbons

**Affiliations:** a Biology Department, Clark University, Worcester, Massachusetts, USA; b Biological Resource Center, Korea Research Institute of Bioscience and Biotechnology, Jeongeup-si, Jeollabuk-do, Republic of Korea; c Food Research Institute, University of Wisconsin—Madison, Madison, Wisconsin, USA; d Department of Bacteriology, University of Wisconsin—Madison, Madison, Wisconsin, USA; e Department of Systems Biotechnology, Konkuk University, Seoul, Republic of Korea; f Department of Food Science, University of Massachusetts, Amherst, Massachusetts, USA; Broad Institute of MIT and Harvard

## Abstract

Acidomyces richmondensis is an extremophilic fungal species found in warm, acidic, and metal-rich environments. To improve upon the existing reference genome, we used PacBio and Illumina sequencing to assemble a highly contiguous 29.3-Mb genome of A. richmondensis FRIK2901.

## ANNOUNCEMENT

Acidomyces richmondensis is an acidophilic dothideomycete capable of growing at a pH as low as 0.1 and optimally at a pH range of 2 to 5. A. richmondensis is part of an extraordinary microbial community present in the warm, acidic, and metal-rich acid mine drainage environment from the Richmond Mine at Iron Mountain in California (1–3). Here, we present a high-quality highly contiguous whole-genome assembly of A. richmondensis FRIK2901. This assembly is a significant upgrade from the existing highly fragmented A. richmondensis reference genome (3) and provides a basis for future genomic and molecular characterization of genes and pathways involved in acid and heavy metal tolerance.

We isolated A. richmondensis FRIK2901 by diluting samples of Richmond Mine water to extinction in growth medium, distributing diluted samples to microtitration plates, and incubating at 37°C for 14 days. A. richmondensis FRIK2901 was grown aerobically at pH 1.0 at 37°C in medium described previously, with some modifications (4). The medium contained (per liter): 20 g FeSO_4_·7H_2_O, 800 mg (NH_4_)_2_SO_4_, 800 mg Ni(NH_4_)_2_ (SO_4_)_2_·6H_2_O, 400 mg KH_2_PO_4_, 160 mg MgSO_4_·7H_2_O, 85 mg Na_2_MoO_4_·2H_2_O, 70 mg ZnCl_2_, 31 mg H_3_BO_3_, 10 mg MnCl_2_·4H_2_O, 10 mg CoCl_2_·6H_2_O, and 0.1% (wt/vol) yeast extract (Becton, Dickinson, Sparks, MD). Genomic DNA was extracted following the method described by Lee et al. (5). A PacBio circular consensus sequence (CCS) library was constructed and sequenced at the University of Wisconsin—Milwaukee on a PacBio RS II platform. A paired-end Illumina library with an average insert size of 450 bp was constructed with the Nextera DNA library preparation kit and sequenced to produce 152-bp reads on an Illumina NextSeq 500 system at ProteinCT (Madison, WI). Raw PacBio sequences were adapter trimmed and error corrected using Canu version 1.6 (6), resulting in a high-quality long-read data set of 112,430 PacBio reads, with an average read length of ∼7 kb. Raw Illumina sequence reads were adapter and quality trimmed using Trim Galore! (7) and then error corrected using Quake (8), resulting in a high-quality short-read data set of 11,603,552 read pairs.

Genome assembly was conducted by first using Canu version 1.6 (6) with the preassembly-improved PacBio data. Next, genome polishing and error correction were performed on the initial PacBio assembly with the preassembly-improved Illumina data using Pilon version 1.21 (9). This hybrid assembly approach resulted in a highly contiguous ∼150× coverage assembly composed of 34 scaffolds, with a GC content of 49.45%, an *N*_50_ value of 1,214,476 bp, a nuclear genome size of 29.25 Mb, and a mitochondrial genome size of 97.70 kb. Genome assembly quality was assessed using BUSCO version 2.0 (10) with the dikarya odb9 data set. Of the 1,312 BUSCO group genes, 97.5% were completely (93.6%) or partially (3.9%) recovered in the A. richmondensis FRIK2901 assembly, indicating a high-quality genome assembly. Gene prediction and annotation were performed using the Funannotate pipeline (11). A total of 8,336 protein-coding genes were predicted, including 306 carbohydrate-active enzymes and 281 peptidases. Interestingly, only 3 secondary metabolite-encoding gene clusters were identified via antiSMASH 3.0 (12). We identified 703 genes in the A. richmondensis FRIK2901 genome that did not have significant BLAST hits (E value cutoff = 1e−6) to the closely related species Hortaea werneckii, the dothideomycete Mycosphaerella graminicola, and the budding yeast Saccharomyces cerevisiae (13). Several of these lineage-specific genes possess domains with potential roles in acid tolerance and heavy metal tolerance.

### Data availability.

This whole-genome shotgun project has been deposited at GenBank under the accession number QVLQ00000000. Raw Illumina and PacBio data have been deposited in the NCBI Sequence Read Archive under the BioProject accession number PRJNA486357.

## References

[1] Baker BJ, Lutz MA, Dawson SC, Bond PL, Banfield JF 2004 Metabolically active eukaryotic communities in extremely acidic mine drainage. Appl Environ Microbiol 70:6264–6271. doi:10.1128/AEM.70.10.6264-6271.2004. 15466574 PMC522060

[2] Baker BJ, Tyson GW, Goosherst L, Banfield JF 2009 Insights into the diversity of eukaryotes in acid mine drainage biofilm communities. Appl Environ Microbiol 75:2192–2199. doi:10.1128/AEM.02500-08. 19201960 PMC2663193

[3] Mosier AC, Miller CS, Frischkorn KR, Ohm RA, Li Z, LaButti K, Lapidus A, Lipzen A, Chen C, Johnson J, Lindquist EA, Pan C, Hettich RL, Grigoriev IV, Singer SW, Banfield JF 2016 Fungi contribute critical but spatially varying roles in nitrogen and carbon cycling in acid mine drainage. Front Microbiol 7:238. doi:10.3389/fmicb.2016.00238. 26973616 PMC4776211

[4] Edwards KJ, Schrenk MO, Hamers R, Banfield JF 1998 Microbial oxidation of pyrite: experiments using microorganisms from an extreme acidic environment. Am Mineral 83:1444–1453. doi:10.2138/am-1998-11-1233.

[5] Lee MK, Park HS, Han KH, Hong SB, Yu JH 2017 High molecular weight genomic DNA mini-prep for filamentous fungi. Fungal Genet Biol 104:1–5. doi:10.1016/j.fgb.2017.04.003. 28427933

[6] Koren S, Walenz BP, Berlin K, Miller JR, Bergman NH, Phillippy AM 2017 Canu: scalable and accurate long-read assembly via adaptive k-mer weighting and repeat separation. Genome Res 27:722–736. doi:10.1101/gr.215087.116. 28298431 PMC5411767

[7] Krueger F 2015 Trim galore! A wrapper tool around Cutadapt and FastQC to consistently apply quality and adapter trimming to FastQ files. https://www.bioinformatics.babraham.ac.uk/projects/trim_galore/.

[8] Kelley DR, Schatz MC, Salzberg SL 2010 Quake: quality-aware detection and correction of sequencing errors. Genome Biol 11:R116. doi:10.1186/gb-2010-11-11-r116. 21114842 PMC3156955

[9] Walker BJ, Abeel T, Shea T, Priest M, Abouelliel A, Sakthikumar S, Cuomo CA, Zeng Q, Wortman J, Young SK, Earl AM 2014 Pilon: an integrated tool for comprehensive microbial variant detection and genome assembly improvement. PLoS One 9:e112963. 25409509 10.1371/journal.pone.0112963 PMC4237348

[10] Simão FA, Waterhouse RM, Ioannidis P, Kriventseva EV, Zdobnov EM 2015 BUSCO: assessing genome assembly and annotation completeness with single-copy orthologs. Bioinformatics 31:3210–3212. doi:10.1093/bioinformatics/btv351. 26059717

[11] Palmer JM 2016 Funannotate: a fungal genome annotation and comparative genomics pipeline. https://github.com/nextgenusfs/funannotate.

[12] Weber T, Blin K, Duddela S, Krug D, Kim HU, Bruccoleri R, Lee SY, Fischbach MA, Muller R, Wohlleben W, Breitling R, Takano E, Medema MH 2015 antiSMASH 3.0—a comprehensive resource for the genome mining of biosynthetic gene clusters. Nucleic Acids Res 43:W237–W243. doi:10.1093/nar/gkv437. 25948579 PMC4489286

[13] Altschul SF, Madden TL, Schaffer AA, Zhang J, Zhang Z, Miller W, Lipman DJ 1997 Gapped BLAST and PSI-BLAST: a new generation of protein database search programs. Nucleic Acids Res 25:3389–3402. doi:10.1093/nar/25.17.3389. 9254694 PMC146917

